# Gli is activated and promotes epithelial-mesenchymal transition in human esophageal adenocarcinoma

**DOI:** 10.18632/oncotarget.22856

**Published:** 2017-12-01

**Authors:** Lei Wang, Joy Q. Jin, Yong Zhou, Ziqiang Tian, David M. Jablons, Biao He

**Affiliations:** ^1^ Department of Thoracic Surgery, Fourth Hospital of Hebei Medical University, Shijiazhuang, Hebei 050011, China; ^2^ Thoracic Oncology Program, Department of Surgery, Helen Diller Family Comprehensive Cancer Center, University of California, San Francisco, CA 94115, USA; ^3^ Department of Respiratory Diseases, Sir Run Run Shaw Hospital, Zhejiang University, Hangzhou, Zhejiang 310016, China

**Keywords:** sonic hedgehog, Gli, epithelial-mesenchymal transition, esophageal adenocarcinoma

## Abstract

Esophageal adenocarcinoma (EAC) accounts for the most esophageal cancer cases in the US, and is notoriously aggressive. This study examines the role of Sonic Hedgehog (SHh)/Gli signaling in the regulation of epithelial-mesenchymal transition (EMT), a process tied to invasion and metastasis, in EAC.

Gli/EMT protein expression levels were examined by western blot in paired EAC patient tissues (*n* = 24) and cell lines (OE19, OE33). Functional analyses were performed (siRNA, treatment with Gli-inhibitor, AKT-inhibitor, and N-Shh recombinant proteins) to investigate SHh/Gli signaling and EMT, cell cycle, and prognostic markers in EAC cell lines. MTS, luciferase reporter, qRT-PCR, western blot, wound healing, and transwell assays were executed to analyze pathway activity, cell migration, and invasion.

Aberrant Gli1/2 expression was found in EAC patient tissues, and was significantly associated with increased EMT and AKT pathway activity. Stimulation of SHh/Gli resulted in EMT signaling, including expression of E-cadherin, N-cadherin, Vimentin, β-catenin, Snail, and Slug, as well as cell cycle progression at mRNA and protein levels in EAC cell lines. Gli inhibition via small molecule administration and siRNA significantly reduced EMT, decreasing cell mobility and invasion. Both Gli and AKT inhibition rescued E-cadherin expression and suppressed AKT phosphorylation.

This study provides evidence for a strong association between aberrant Gli1/2 expression and AKT/EMT markers in EAC; activated SHh/Gli signaling may be a critical component in promoting cell survival, metastases, and resistance to chemotherapy, and represents a promising avenue to target tumor proliferation and mobility.

## INTRODUCTION

Esophageal carcinoma is one of the leading causes of cancer-related deaths worldwide, with low 5-year survival rates at approximately 12-20% [1, 2]. Esophageal adenocarcinoma (EAC) is one of the most common subtypes of esophageal cancer [1, 3]. Patients suffer from poor prognoses and high incidence of recurrence and metastasis, even following positive response to traditional therapeutic strategies including surgery, chemotherapy, and radiotherapy [4, 5]. Thus, more meaningful treatment strategies are desperately needed to target the mechanisms leading to tumor invasion and metastasis in EAC.

The epithelial-mesenchymal transition (EMT) is a process in which cells lose their cell-cell adhesive properties and gain migratory and invasive potential; it is essential for events in embryonic development, wound healing, fibrosis, cancer progression, and metastasis [6–8]. EMT involves multiple complex changes in the distribution and function of proteins, including E-cadherin, an adhesive protein inactivated in numerous cancers [8]. This process is regulated by a number of converging signaling cascades, including the Sonic Hedgehog (SHh) pathway, and confers critical traits required for seeding metastasis and developing stem cell properties that allow new cancer cell colonies to be launched [9]. Acquisition of EMT features has been associated with poor prognosis and chemotherapeutic resistance—accordingly, further knowledge can improve our understanding of tumor recurrence and metastasis to identify potential therapeutic targets in EAC [10–13].

Activation of SHh signaling has been implicated in the tumorigenesis and metastasis of various cancers [14–26]. The canonical cascade is initiated by Patched (Ptch) and Smoothened (Smo); Sonic Hedgehog ligand (Shh) binding to Ptch allows release of Smo, causing active full-length Gli to enter the nucleus and activate transcription of target genes, including Gli1, Ptch1, and Cyclin D1, in a context- and cell-type dependent manner [27]. Among the Gli family of transcription factors (TFs), Gli1 and Gli2 are considered activators, while Gli3 serves as a repressor [27]. Non-canonical Gli activation independent of Shh activation has also been noted in many cancer cells types, owing to stimulation by other oncogenic signaling pathways such as transforming growth factor β (TGF-β), epidermal growth factor receptor (EGFR), RAS, and AKT/PI3K [28–32]. As Gli TFs constitute the final effectors of the SHh pathway, and are implicated in multiple other oncogenic signaling cascades, they represent an important downstream target for potential cancer therapeutics. Efforts have been made to develop inhibitors of the SHh pathway, including Vismodegib, a Smo inhibitor, as well as GANT-61, a Gli inhibitor that regulates Gli-dependent transcription, to promote anti-cancer activity [33, 34]. In our lab, we have also developed a novel Gli inhibitor (Gli-i) that specifically blocks Gli1 and Gli2 transcriptional activity with significant efficacy [35].

However, the relationship between EMT and SHh/Gli activation has not previously been studied in EAC, and existing data from other solid tumor types are controversial. While some studies have shown that SHh/Gli inhibition block EMT, the exact mechanisms have not been elucidated. In melanoma and pancreatic cancers, results suggest the role of Gli in facilitating cancer migration and invasion by regulation of E-cadherin [36, 37]. On the other hand, another conflicting report proposes the inhibition of Gli in promoting the same EMT characteristics in pancreatic cancer [38]. In lung squamous cell cancer (SCC), Gli expression is inversely correlated to that of E-cadherin [39]. Studies in melanoma and hepatocellular carcinoma have linked Gli1 to vascular/capsular invasion, advanced tumor stage, and upregulation of matrix metalloprotease (MMP)-2 and MMP-9, while siRNA silencing of Gli1 successfully reduced invasion and increased E-cadherin expression [40, 41]. Our lab recently studied upregulated signaling in lung cancer, investigating Gli1’s inverse correlation with E-cadherin; inhibition of the SHh pathway upregulates E-cadherin expression and suppresses lung cancer cell migration [39].

In this study, aberrant Gli activation was studied in both EAC tissue samples and cell lines. Gli and EMT markers were found to be inversely correlated, and inhibition of the former minimized migration and invasion of EAC cells. Gli suppression induced upregulated E-cadherin expression and downregulated phosphorylated AKT, suggesting Gli may be critical for the metastasis and recurrence of esophageal adenocarcinomas.

## RESULTS

### Gli is upregulated in EAC tissue samples, and correlates with EMT and AKT pathway markers

**Figure 1:**
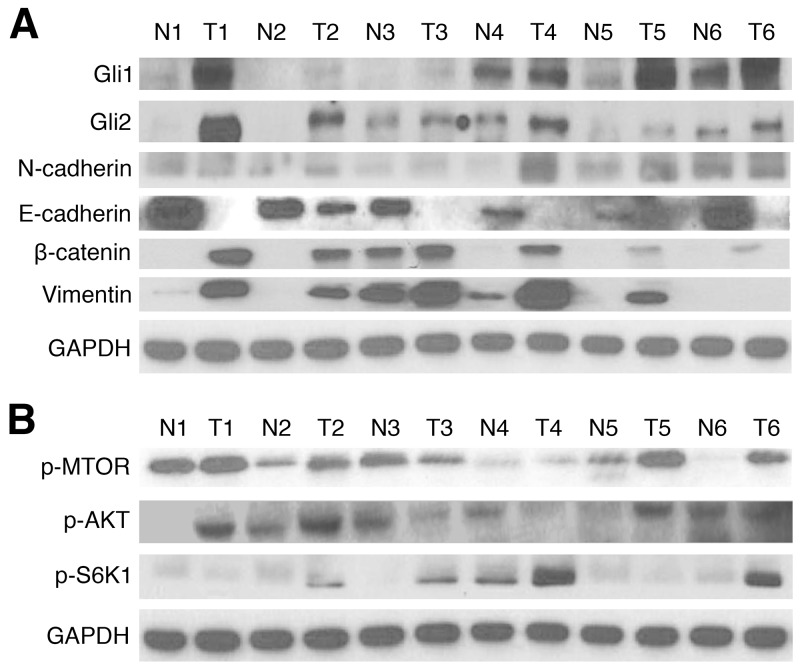
Gli1/2, EMT, and AKT pathway markers are upregulated in EAC tissue samples **(A)** Western blots of Gli1 and Gli2 protein expression in 24 matched pairs of esophageal tumor (T) and normal (N) tissues. GAPDH served as a loading control; results of 6 numbered, representative sample pairs are shown here. EMT markers (E-cadherin, N-cadherin, Vimentin, and β-catenin) were also examined. **(B)** Western blots of AKT pathway protein (p-MTOR, p-AKT, and p-S6K1) expression in 24 matched pairs of esophageal T and N tissues. GAPDH served as a loading control; results of 6 representative sample pairs are shown.

Previous studies have suggested presence of increased Gli signaling components in EAC, but have not investigated the relationship between aberrant Gli activation and EMT. Protein expression of Gli1, Gli2, and key EMT pathway markers was first examined in 24 matched EAC and normal patient tissue samples from the Thoracic Oncology Program at University of California, San Francisco (UCSF). In western blot analysis, 91.7% (22/24) and 87.5% (21/24) of tumor samples showed higher Gli1 and Gli2 expression than in paired normal tissue, respectively (Figure 1A, Supplementary Figure 1A). Overexpression of N-cadherin, a biomarker associated with increased EMT [42], was observed in 83.3% (20/24) in tumor samples, while a loss of E-cadherin was seen in 95.8% (23/24) of tumor tissues. Activation of β-catenin and vimentin, both correlated with EMT invasive and proliferative characteristics [43, 44], was observed in 83.3% (20/24) of the patient sample cohort. AKT pathway proteins were subsequently analyzed via western blot (Figure 1B, Supplementary Figure 1B); activation of m-TOR, p-AKT, and p-S6K1 was observed in 87.5% (21/24), 75% (18/24), and 83.3% (20/24) of the tumor tissue samples, respectively. M-TOR activation has previously been shown to induce overexpression of downstream effectors, including p-S6K1 (activated S6K1); both factors have been linked to crosstalk with Gli signaling, and are associated with enhanced aggression [45, 46]. On the other hand, p-AKT plays an important role in tumorigenesis and resistance to therapy in EAC [47]. Thus, our results demonstrate Gli1 and Gli2 hyperactivation in EAC patient tissue samples, with strong correlations to 7 pathway markers implicated in upregulated EMT signaling.

### Gli inhibition and siRNA knockdown reduce EMT and cell cycle activity in EAC cell lines

To further understand the role of Gli in affecting EMT and cell cycle activity, two EAC cell lines, OE19 and OE33, were subjected to cell viability, reporter, and qRT-PCR analyses. Inhibition of Gli activity was achieved by treating cells with a Gli inhibitor (Gli-i) developed by our lab, which has been shown to specifically target Gli1 and Gli2 transcriptional activity [35]. Stimulation of the SHh/Gli pathway was achieved by applying recombinant Shh proteins (N-Shh) to the cultured cells.

**Figure 2:**
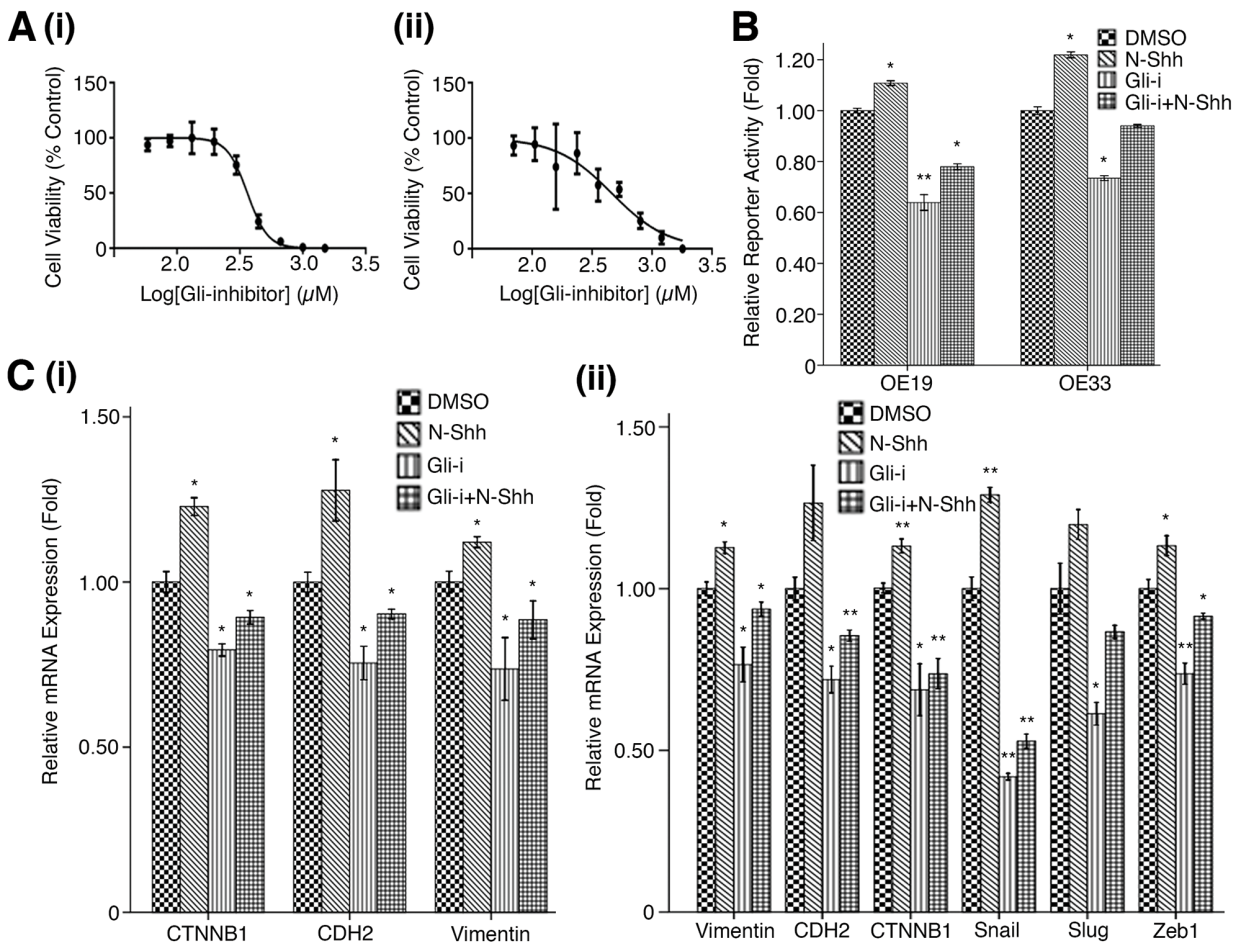
Gli inhibition reduces EMT activity in EAC cell lines **(A)** MTS cell viability assay in (i) OE19, and (ii) OE33 EAC cell lines. Cells were subjected to a serial dilution of Gli-i with DMSO control over a 3-day period, yielding IC_50_ values of 363.2 nM and 464.3 nM, respectively. **(B)** Luciferase reporter assay in OE19 and OE33, treated with DMSO (control), N-Shh (0.5 mg/mL), Gli-i (500 nmol/L), or Gli-i (500 nmol/L) and N-Shh (0.5 mg/mL) stimulation, for 24 hours. Results are expressed as fold activity, i.e. the ratio of luciferase activity induced in Gli-transfected cells relative to basal luciferase activity in control transfected OE19/33 cells (p-values of < 0.05, < 0.01, or < 0.001 were indicated as ^*^, ^**^, or ^***^, respectively). **(C)** qRT-PCR expression levels of EMT markers in (i) OE19 (CTNNB1, CDH2, Vimentin), and (ii) OE33 (Vimentin, CDH2, CTNNB1, Snail, Slug, Zeb1). Cells were treated with DMSO, N-Shh, Gli-i, or Gli-i + N-Shh prior to RNA extraction according to the conditions described above (p-values of < 0.05, < 0.01, or < 0.001 were indicated as ^*^, ^**^, or ^***^, respectively).

MTS assays using a serial dilution of Gli-i (with DMSO as vehicle control) in both OE19 and OE33 EAC cell lines yielded IC_50_ values of 363.2 nM and 464.3 nM, respectively (Figure 2A). Results suggest Gli inhibition may drastically reduce EAC cell viability; luciferase reporter assays were then performed to measure Gli-mediated transcriptional activity in both cell lines (Figure 2B). As expected, N-Shh stimulation of cells significantly increased reporter activity in OE19 and OE33 (p < 0.05), while Gli-i treatment of transfected cells showed over 30% less relative reporter activity in both cell lines. When subjected to both Gli-i and N-Shh treatment, OE19 and OE33 continued to demonstrate decreased Gli transcriptional activity. qRT-PCR analyses of EMT markers in cells treated in the same conditions (DMSO, N-Shh, Gli-i, or Gli-i + N-Shh) produced consistent results (Figure 2C). While N-Shh stimulation increased relative mRNA expression of CTNNB1 (gene coding for β-catenin), CDH2 (gene coding for N-cadherin), Vimentin, Snail, Slug, and Zeb1, administration of Gli-i in OE19 and OE33 cells significantly reduced mRNA expression levels of all markers tested (p < 0.05). All six genes (CTNNB1, CDH2, Vimentin, Snail, Slug, and Zeb1) are positively associated with EMT activity; Snail, Slug, and Zeb1 function to repress E-cadherin, which typically adheres adjacent epithelial cells in carcinomas, leading to increased metastasis and EMT [48, 49].

**Figure 3:**
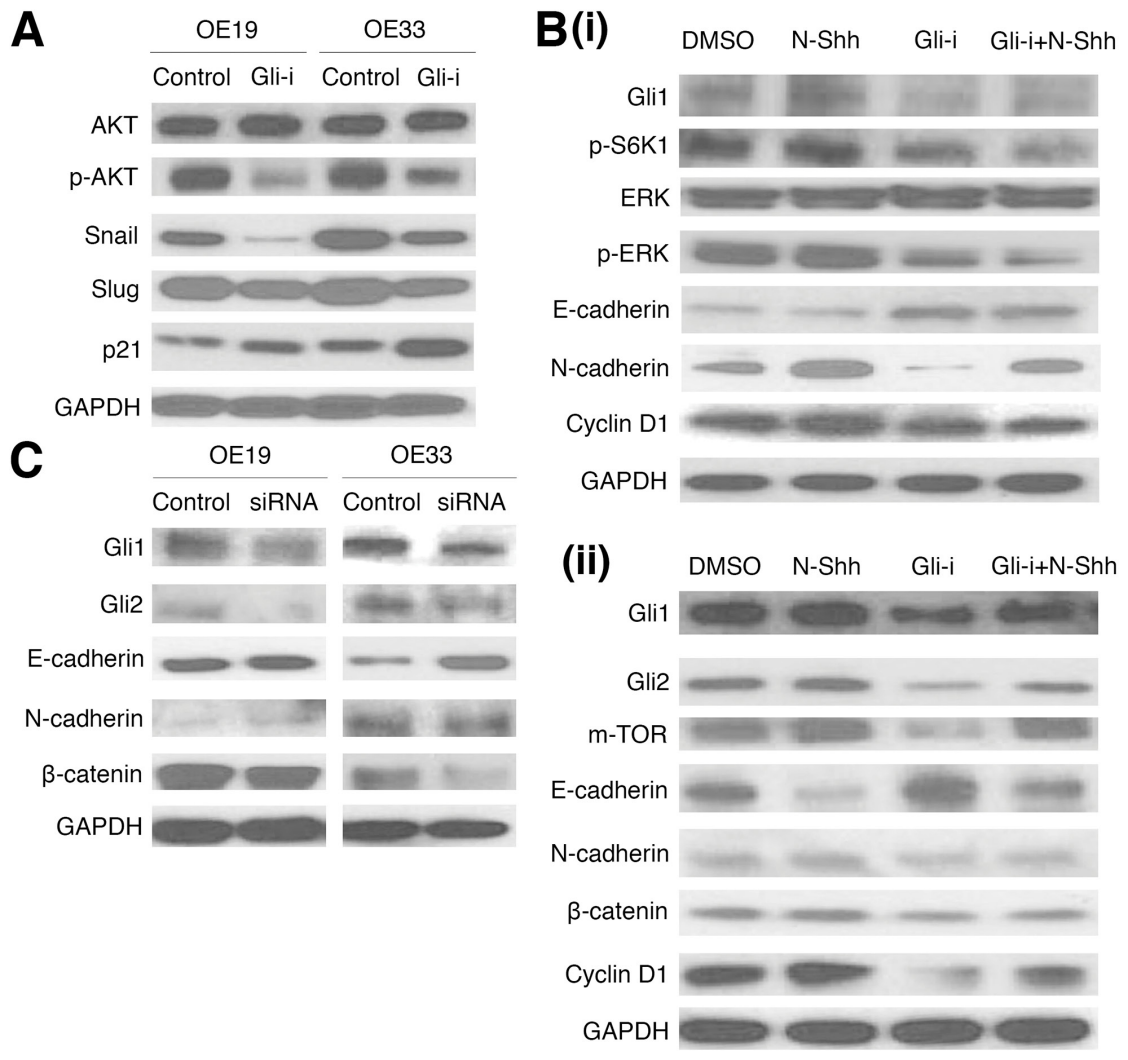
Gli inhibition and siRNA knockdown reduce EMT and cell cycle activity in EAC cell lines **(A)** Western blots of OE19 and OE33 cells, pretreated with either DMSO (control) or Gli-i (500 nmol/L). Expression of AKT, p-AKT, Snail, Slug, and p21 was examined, with GAPDH serving as a loading control. **(B)** Western blots of EMT and cell cycle signaling markers in (i) OE19 (Gli1, p-S6K1, ERK, p-ERK, E-cadherin, N-cadherin, Cyclin D1), and (ii) OE33 (Gli1, Gli2, m-TOR, E-cadherin, N-cadherin, β-catenin, Cyclin D1), with GAPDH as a control. Cells were pretreated with DMSO, N-Shh (0.5 mg/mL), Gli-i (500 nmol/L), or Gli-i (500 nmol/L) with N-Shh (0.5 mg/mL) stimulation prior to protein extraction. **(C)** Western blots of OE19 and OE33 cells, subjected to siRNA treatment. Cells were given control siRNA (100 nM) or Gli1 + Gli2 siRNA (100 nM each) for 48 hours prior to total protein extraction. Expression of Gli1, Gli2, E-cadherin, N-cadherin, and β-catenin was examined, with GAPDH serving as an internal control.

To determine protein expression patterns of EMT and related markers following Gli-i treatment, western blots were then conducted in OE19 and OE33 (Figure 3). Gli inhibition is associated with decreased p-AKT expression (normalized against total AKT protein levels), as well as positive correlation with p21, and negative correlation with Snail and Slug (Figure 3A). The relationship of Gli-i treatment with increased p21 is especially promising given that p21, a Cyclin-dependent kinase (Cdk) inhibitor, promotes cell cycle arrest and negatively regulates cell cycle activities [50]. Additionally, Cyclin D1 showed a decrease in expression level following Gli-i administration, suggesting a decrease in TGF-β mediated cell migration (Figure 3B) [51]. Further examination of p-ERK revealed decreased expression (normalized against total ERK protein levels) following Gli-i incubation, even with N-Shh stimulation (Figure 3B). Activation of ERK is required for TGF-β1 induced EMT [52]. EMT markers, including E-cadherin, N-cadherin, and β-catenin, showed results consistent with Figure 1; Gli-i induced a decrease in E-cadherin and increase in expression of the latter two markers.

In addition, functional analyses with siRNA knockdown of both Gli1 and Gli2 yielded western blot data consistent with earlier parts of the figure, showing decreased Gli1, Gli2, and EMT activity (as measured by E-cadherin, N-cadherin, and β-catenin) in both OE19 and OE33 cell lines (Figure 3C). Taken together, these results strongly suggest that inhibition of Gli signaling may suppress EMT and cell cycle progression in EAC.

### Gli signaling promotes cell migration and invasion in EAC

**Figure 4:**
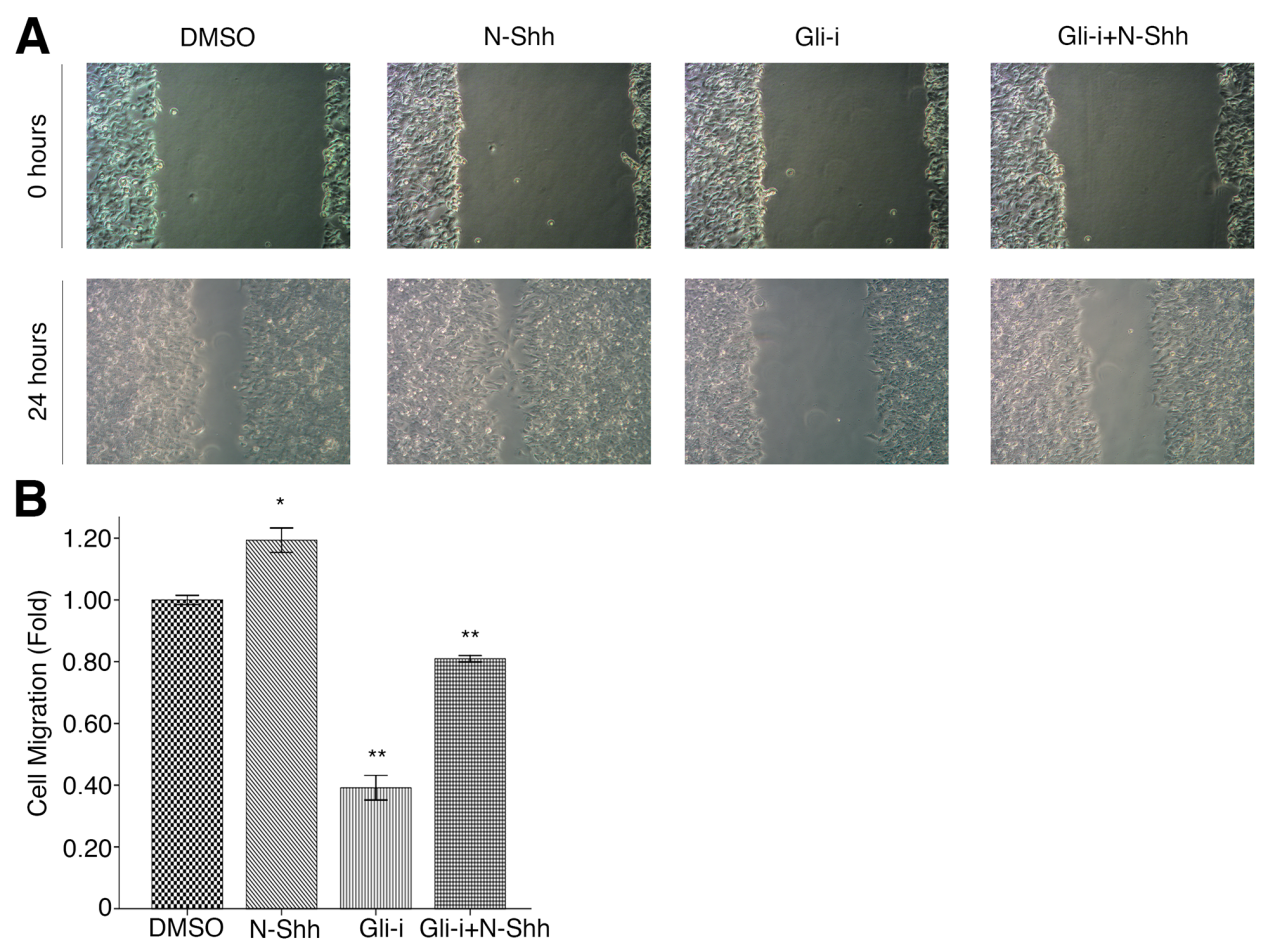
Gli signaling promotes cell migration in EAC **(A)** Wound healing assays of OE33 EAC cells, treated with DMSO (control), N-Shh (0.5 mg/mL), Gli-i (500 nmol/L), or Gli-i (500 nmol/L) with N-Shh (0.5 mg/mL) stimulation. Representative pictures taken at 0 and 24 hours under a light microscope are shown (100x). **(B)** Quantification of wound healing assays, expressed as fold change in comparison to the control group (p-values of < 0.05, < 0.01, or < 0.001 were indicated as ^*^, ^**^, or ^***^, respectively).

**Figure 5:**
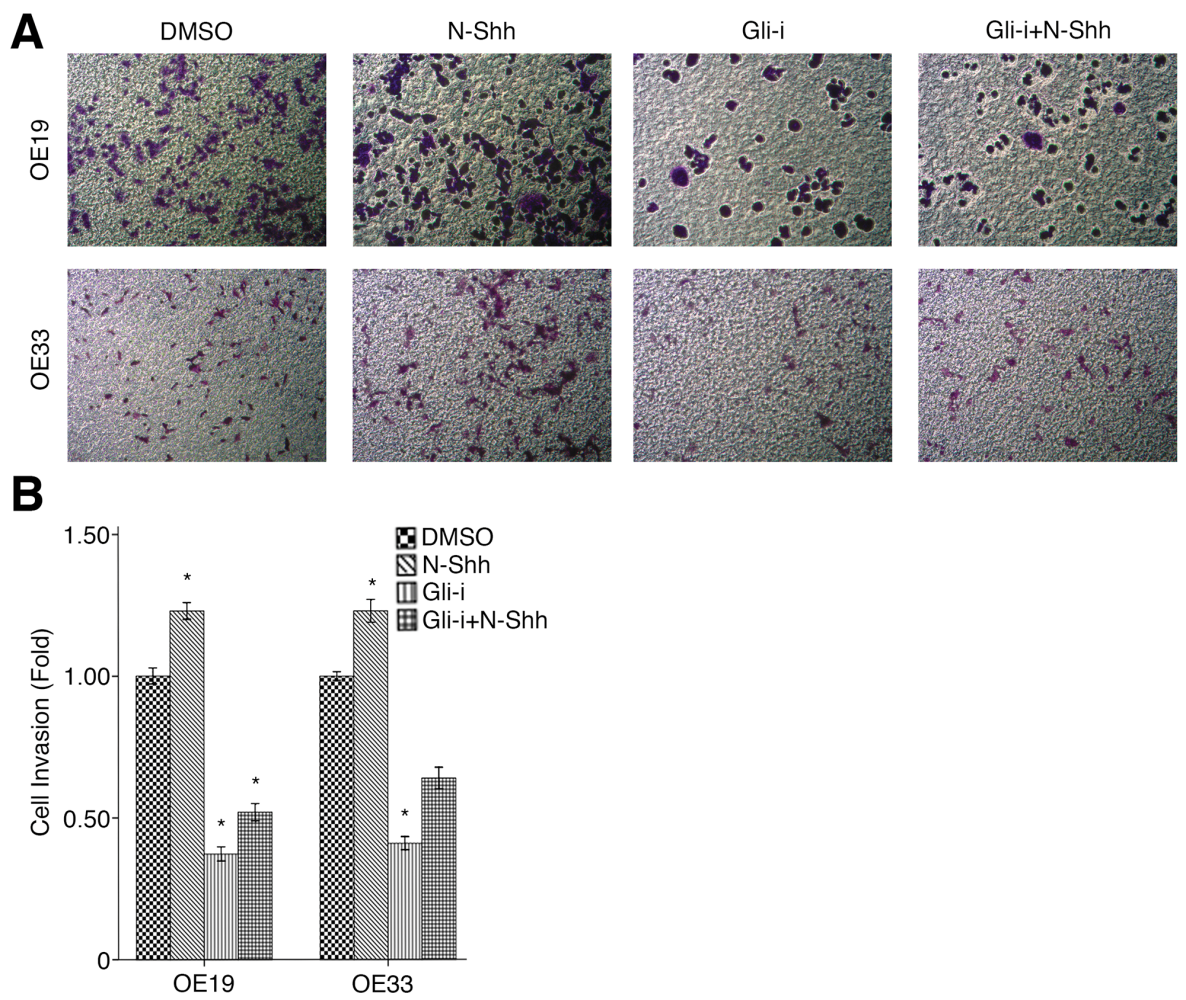
Gli signaling promotes cell invasion in EAC **(A)** Transwell invasion assay of EAC OE19 and OE33 cells, treated with DMSO (control), N-Shh (0.5 mg/mL), Gli-i (500 nmol/L), or Gli-i (500 nmol/L) with N-Shh (0.5 mg/mL) stimulation. Representative images captured by a light microscope are shown (100x). **(B)** Quantification of transwell invasion assays, expressed as fold change in comparison to the control group (p-values of < 0.05, < 0.01, or < 0.001 were indicated as ^*^, ^**^, or ^***^, respectively).

To assess the effects of Gli signaling on cell migration and invasion in EAC, wound healing and transwell assays were conducted in OE19 and OE33 cell lines, subjected to treatment with DMSO (vehicle control), N-Shh, Gli-i, and Gli-i + N-Shh in the same manner as above. Over the course of 24 hours, OE33 cells exhibited significantly slower migration when treated with Gli-i (Figure 4A). Cells stimulated by N-Shh demonstrated greater mobility during the same period, although such cells still migrated slower than the DMSO control when Gli-i was administered (Figure 4B). Furthermore, in transwell invasion assays, both OE19 and OE33 demonstrated decreased invasion by approximately 50% following Gli-i treatment (Figure 5A, 5B). Consistent with data in earlier figures, these results indicate that Gli signaling may promote both cell migration and invasion through loss of E-cadherin in EAC.

### AKT inhibition reduces EMT and cell cycle activity in EAC cell lines

**Figure 6:**
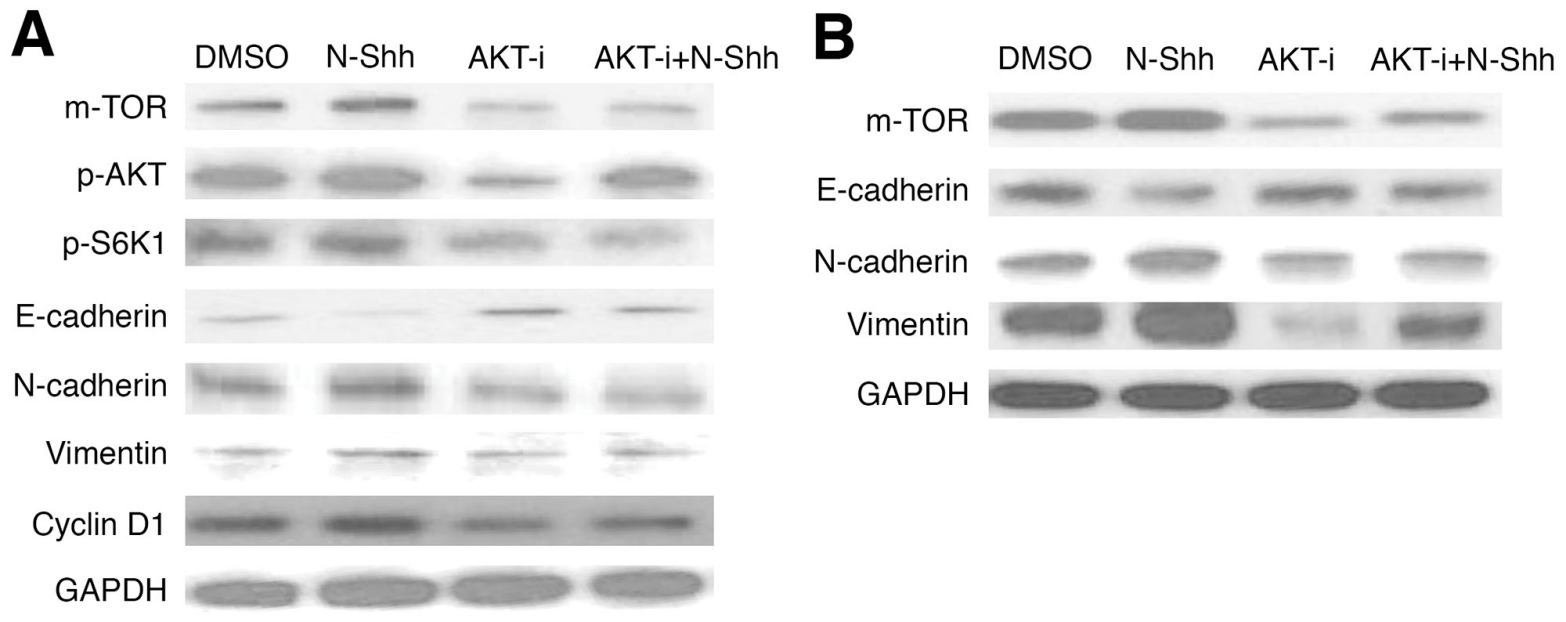
AKT inhibition reduces EMT and cell cycle activity in EAC cell lines Western blots of EMT and cell cycle signaling markers in **(A)** OE19 (m-TOR, p-AKT, p-S6K1, E-cadherin, N-cadherin, Vimentin, Cyclin D1), and **(B)** OE33 (m-TOR, E-cadherin, N-cadherin, Vimentin), with GAPDH as a loading control. Cells were pretreated with DMSO, N-Shh (0.5 mg/mL), AKT-i (1 μmol/L), or AKT-i (1 μmol/L) with N-Shh (0.5 mg/mL) stimulation prior to protein extraction.

Further examination on AKT signaling was conducted in EAC cell lines, following initial investigation (Figures 1-3). OE19 and OE33 EAC cells were administered MK2206, an AKT inhibitor (AKT-i), and analyzed by western blot to determine protein levels of relevant pathway and EMT markers after 30 hours of incubation. In OE19 (Figure 6A, Supplementary Figure 2A), AKT-i treated cells exhibited decreased m-TOR, p-AKT, and p-S6K1, as well as inhibited EMT activity (increased E-cadherin, decreased N-cadherin and Vimentin); Cyclin D1 levels were lower in both AKT-i and AKT-i + N-Shh stimulated cells. Consistent with earlier results, AKT inhibition yielded decreased m-TOR, N-cadherin, and Vimentin, alongside increased E-cadherin levels in OE33 (Figure 6B, Supplementary Figure 2B). N-Shh has previously been shown to promote EMT, cell migration, and invasion [53]. As with Gli-i, N-Shh used in combination with AKT-i rescues the effects induced by the latter, suggesting that SHh/Gli signaling may regulate EMT via the AKT pathway in EAC. Thus, inhibition of AKT and Gli signaling may serve as a promising avenue for decreased EMT and cell cycle activity, both of which are linked to greater aggression and viability in EAC.

## DISCUSSION

SHh/Gli signaling plays an important role in solid malignancies; downstream pathway effectors are tied to EMT, and many serve as potential therapeutic targets in the prevention of tumor aggression and metastasis. This study provides strong preliminary evidence that *in vitro* Gli inhibition impairs the migratory, invasive, and proliferative properties of EAC cells by reducing EMT. EAC tissue specimens from patients demonstrated inverse correlations between Gli1/2 and key EMT markers, including cell adhesion protein E-cadherin, in addition to a positive correlation with N-cadherin, Vimentin, and β-catenin, all three of which are linked to increased EMT [42-44, 54]. Data also indicated strong relationships to increased m-TOR, p-S6K1, and phosphorylated AKT, which play important roles in tumorigenesis, resistance to therapy, and cancer aggression [45–47]. After demonstrating that high levels of SHh/Gli activity were linked to the EMT pathway and its regulators, functional study showed the important effects of Gli-inhibition on reversing these expression patterns. Administration of a novel Gli-i small molecule, in addition to siRNA knockdown and AKT-i treatment, decreased expression of both proteins involved in cell cycle progression and thus proliferation, as well as markers responsible for the characteristic properties of EMT. Furthermore, when used in combination with Gli-i and AKT-i, N-Shh can rescue the effects of such inhibitors, suggesting that SHh/Gli signaling may regulate EMT via the AKT pathway in EAC. These findings were corroborated by results in both wound healing and transwell invasion assays following Gli-i treatment. Investigation via western blot and qRT-PCR paint a more complete picture of potential mechanisms responsible for declining migratory capabilities following SHh signal suppression.

EMT regulation is a sophisticated process; the SHh/Gli pathway may interact at various levels, including direct transcription of Snail, an EMT marker also investigated in this study [53]. Crosstalk between SHh and TGF-β signaling may play an important role in EMT regulation, while at the protein level, interactions between Gli and β-catenin at cell-cell junctions may directly contribute to cell mobility [53]. Thus, the molecular mechanism behind SHh pathway regulation of EMT in EAC requires further exploration.

Upstream of Gli, Vismodegib, a Smo inhibitor preventing activation of SHh target genes, has already been approved for basal cell carcinoma treatment, and is looking to expand its use to other cancers [55, 56]. While Vismodegib shows promise for reducing tumor size and lengthening progression free survival times, two mutations (D477G, E518K) conferring resistance mechanisms have already been identified in relapsed patients [57, 58]. Second generation Smo inhibitors, including Itraconazole and arsenic trioxide, both inhibit the growth of medulloblastoma and basal cell carcinoma, prolonging survival of mice with intracranial drug-resistant Smo [59, 60]. In addition to development of upstream SHh inhibitors, we propose that downstream Gli inhibition may prove even more effective than current Smo targeting that is currently in development. Gli suppression has the potential to circumvent upstream resistance mechanisms that have posed challenges thus far, given evidence of *(1)* non-canonical Gli activity independent of SHh in cancer, and *(2)* Gli as a target of other converging oncogenic signaling pathways [61–66]. While further research is still required, including additional patient tissue studies, Gli inhibition is a promising area for further investigation and potential drug development, owing to its close connection to EMT and regulation of tumor mobility in EAC.

This study provides evidence for aberrant upregulation of the Gli signaling pathway and a strong association between expression of Gli versus AKT, EMT, and cell cycle checkpoint markers in EAC. These findings suggest that activated SHh/Gli signaling may be a critical component in promoting cell survival, metastases, and resistance to chemotherapy. Inhibition of Gli and AKT pathway activity may thus serve as a potential therapeutic strategy for the treatment of human esophageal adenocarcinoma.

## MATERIALS AND METHODS

### Tissue specimens

Tissue specimens were collected from 24 patients who underwent surgical resection for esophageal adenocarcinoma (EAC) at the Thoracic Oncology Program at the University of California, San Francisco (UCSF). The study was approved by the Committee on Human Research (CHR approval number: H8714-11647-10) at UCSF; written, informed consent was obtained from each patient before specimen collection. These tissue samples were snap-frozen in liquid nitrogen immediately after resection and kept at -170°C before use.

### Cell culture and drug treatment

Human esophageal adenocarcinoma (EAC) cell lines OE19 and OE33 were purchased from American Type Culture Collection (ATCC, Manassas, VA, USA) and cultured in RPMI 1640 (Life Technologies, Carlsbad, CA, USA), supplemented with 10% fetal bovine serum (FBS) and antibiotics. Cells were seeded one day before treatment with Gli inhibitor (Gli-i) or AKT inhibitor MK-2206 2HCI (AKT-i) (Selleck Chemicals, Houston, TX, USA), with or without N-Shh recombinant proteins (eBioscience, San Diego, CA, USA), and incubated for 30 hours, with DMSO vehicle as a control. Cells were then cultured for various periods of time prior to harvesting for subsequent analyses, including western blot, qRT-PCR, proliferation, migration, and invasion assays.

### Protein extraction and western blot

OE19 and OE33 cells were pretreated with DMSO (control), N-Shh (0.5 mg/mL), Gli-i (500 nmol/L), or AKT-i (1 μmol/L), with or without subsequent stimulation by N-Shh (0.5mg/mL) for 24 hours. Total protein was then extracted from cell lines using M-PER Mammalian Protein Extraction Reagent (ThermoFisher Scientific, Waltham, MA, USA) and Complete Protease Inhibitor Cocktails (Roche, Lewes, UK), according to manufacturers’ protocols. Protein concentrations were determined using the Pierce BCA Protein Assay Kit (ThermoFisher Scientific), with 10 μg per sample run on 4-20% gradient SDS-polyacrylamide gels before transfer to Immobilon-P membranes (Millipore, Billerica, MA, USA). Nitrocellulose membranes were blocked in 5% nonfat milk and probed with the primary antibodies overnight at 4°C. The membranes were incubated with appropriate secondary antibodies, followed by detection using an ECL blotting analysis system. Antibodies applied to detect protein expression are as follows: Gli1 (1:100), Cyclin D1 (1:200), Snail (1:100), and Slug (1:150) (Santa Cruz Biotechnology, Dallas, TX, USA); Gli2 (1:150) and phosphorylated m-TOR (1:200) (Abcam, Cambridge, UK); N-Cadherin (1:200), E-cadherin (1:100), and β-catenin (1:200) (EMD Millipore, Hayward, CA, USA); Vimentin (1:200), p-AKT (1:200), AKT (1:200), p-ERK (1:200), and ERK (1:200) (Cell Signaling, Danvers, MA, USA); and GAPDH (1:500) (Sigma-Aldrich, St. Louis, MO, USA).

### Luciferase reporter assay

To measure Gli-mediated Shh transcriptional activity, luciferase reporter constructs, 8x wild-type Gli binding site (8x Gli^wt^ Luc) or 8x mutant Gli binding site (8x Gli^mut^ Luc) plasmids, and human Gli1 and Gli2 expression vectors (pcDNA3.1-Gli1/2) were co-transfected into EAC cell lines OE19 and OE33 in 24-well plates. The Renilla luciferase pRL-TK plasmid (Promega, Madison, WI, USA) was co-transfected to normalize for transfection efficiency according to manufacturer’s protocol. All transfections were performed using Lipofectamine2000 (Invitrogen, Carlsbad, CA, USA) in accordance with the manufacturer’s instructions. OE19 and OE33 cells were pretreated with DMSO, N-Shh (0.5 mg/mL), and/or Gli-i (500 nmol/L) for 30 minutes. After 24 hours, cells were lysed and subjected to the luciferase assays described above. Results are expressed as fold induction, which is the ratio of luciferase activity induced in Gli-transfected cells relative to basal luciferase activity in control transfected cells.

### RNA extraction and qRT-PCR

Total RNA was isolated from OE19 and OE33 cultured cells using an RNeasy kit (Qiagen, Hilden, Germany). Genomic DNA contamination was eliminated by DNase I treatment, and reverse transcription was conducted with 500 ng RNA using the iScript cDNA synthesis kit (Bio-Rad, Hercules, CA, USA). The resulting cDNA were analyzed with real-time quantitative reverse transcription polymerase chain reaction (qRT-PCR) using Gene Expression Assays in a 7900 Real-Time PCR System (Applied Biosystems, Foster City, CA, USA) for 40 cycles (96°C for 15 seconds and 60°C for 1 minute). Expression of Vimentin, CDH2, CTNNB1, Snail, Slug, Zeb1, and GAPDH was quantified using commercially available primer and probe sequences (Applied Biosystems), and Relative Quantification Software (Applied Biosystems). Gene expression was normalized to GAPDH expression; experiments were performed in triplicate and repeated three times.

### siRNA transfection

OE19 and OE33 cells were plated in six-well plates with fresh media without antibiotics for 24 hours before transfection. Transfection was performed using Lipofectamine2000 (Life Technologies, Carlsbad, CA) according to manufacturer’s protocol, with siRNAs targeting Gli1 (Assay ID 107670) and Gli2 (Assay ID 109640) at a total concentration of 50 nmol/L (Life Technologies). The efficiency of siRNA transfection was evaluated by western blot.

### MTS survival, wound healing, and transwell invasion assays

In MTS survival assays, treated cells were plated in 96-well plates at a density of 500-1000 cells/well, with medium changed daily. Logarithmically growing cells were treated with increasing doses of Gli-i or AKT-i, and a DMSO vehicle control for 3 days. Cells were subsequently assessed for cell viability using CellTiter-Glo Luminescent Cell Viability Assay reagent (Promega, Madison, WI, USA) according to manufacturer’s instructions. Luminescence was measured using a GloMax-96 Microplate Luminometer (Promega), and percent cell survival was calculated based on the reading of untreated cells as 100% using GraphPad Prism 6.0 software to generate dose-response curves and IC_50_ values. All samples were conducted in triplicate, and data were standardized to that of DMSO treated cells.

In migration assays, four wounds were made in each treatment condition, and cell migration was determined by the average differences in distance between 0 and 24 hours following treatment. Transwell invasion assays (ThermoFisher Scientific) were performed according to manufacturer’s protocols. All experiments were conducted more than three times, and representative results were included in the text.

### Statistical analysis

Two-sided student *t*-tests were performed for wound healing (migration) and transwell (invasion) assay analyses, while ANOVA and Scheffe’s tests were used to determine significance in luciferase reporter and qRT-PCR data. P-values of < 0.05, < 0.01, or < 0.001 were indicated as ^*^, ^**^, or ^***^, respectively, in figures.

## SUPPLEMENTARY MATERIALS FIGURES



## Abbreviations

AKT-i: AKT inhibitor
Cdk: Cyclin-dependent kinase
EAC: esophageal adenocarcinoma
EGFR: epidermal growth factor receptor
EMT: epithelial-mesenchymal transition
Gli-i: Gli inhibitor
MMP: matrix metalloprotease
N: normal
P: phosphorylated
Ptch: Patched
q-RTPCR: real-time reverse transcription polymerase chain reaction
SCC: squamous cell cancer
SHh: Sonic Hedgehog (pathway)
Shh: Sonic Hedgehog (ligand)
Smo: Smoothened
T: tumor
TF: transcription factor
TGF-β: transforming growth factor β
UCSF: University of California, San Francisco
