# Supplementary material for: Gli is activated and promotes epithelial-mesenchymal transition in human esophageal adenocarcinoma
Source: Oncotarget. 2017 Dec 1;9(1):853–65. doi: 10.18632/oncotarget.22856 (PMC5787518; doi:10.18632/oncotarget.22856)
Supplement: Supplementary file 1 [file oncotarget-09-853-s001.pdf]

Gli is activated and promotes epithelial-mesenchymal transition in human esophageal adenocarcinoma

SUPPLEMENTARY MATERIALS

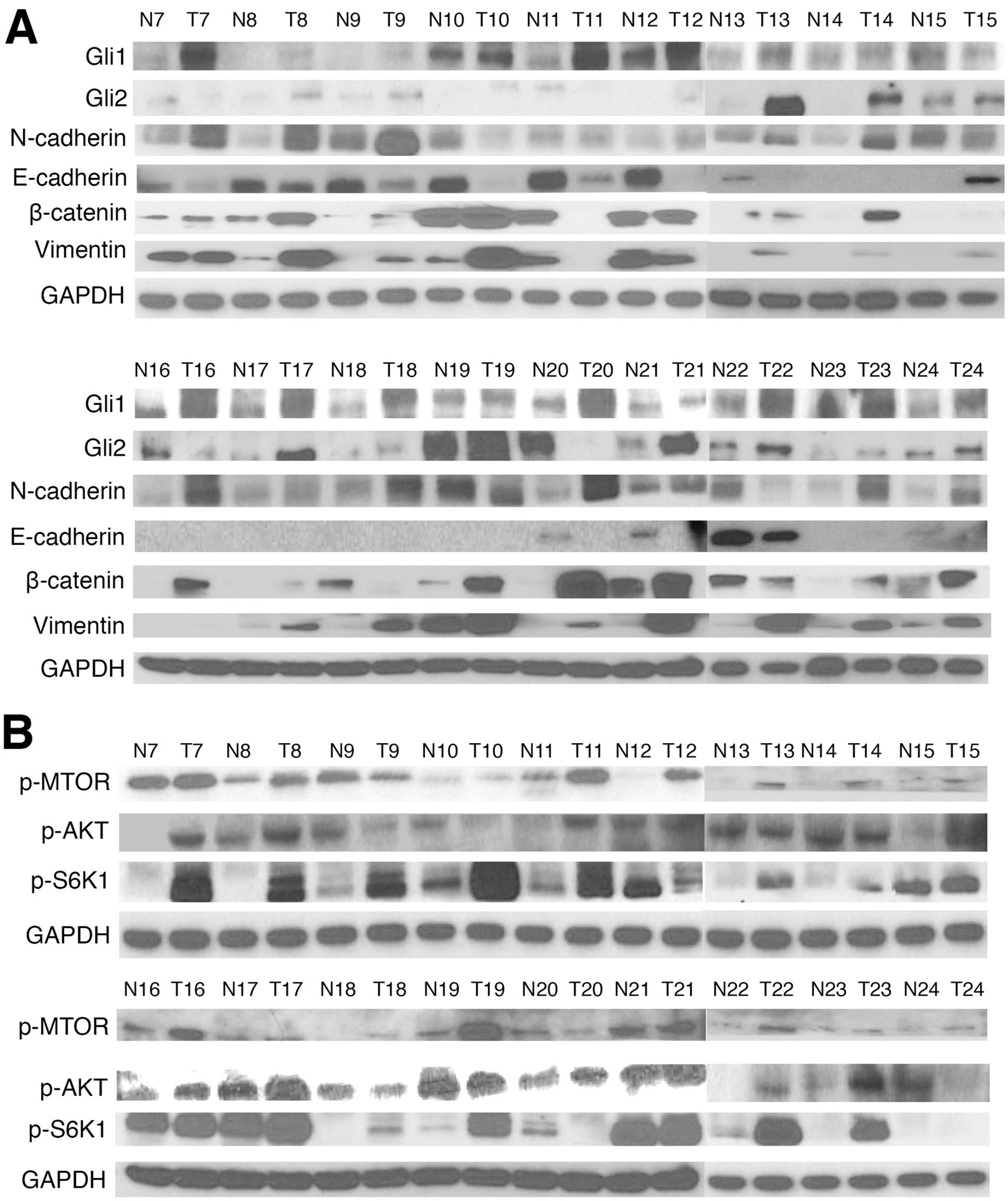

Supplementary Figure 1: Western blots of the remaining 18 out of 24 total matched pairs of esophageal N and T patient tissues, supplementing the 6 representative pairs shown in Figure 1. **(A)** Gli1 and Gli2, with GAPDH as a loading control. **(B)** AKT pathway proteins (p-MTOR, p-AKT, and p-S6K1), with GAPDH as a loading control.

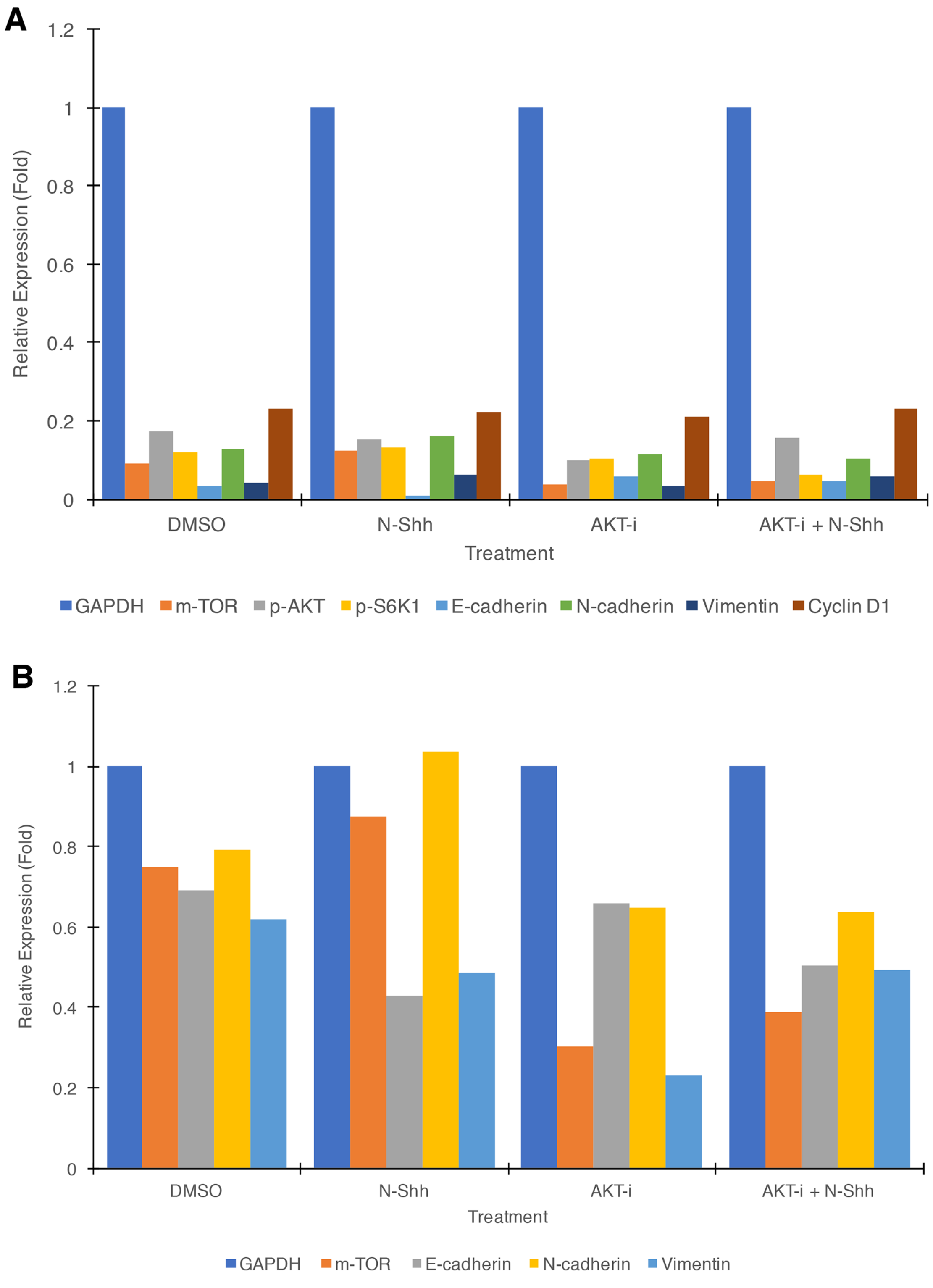

Supplementary Figure 2: Quantification of western blots shown in Figure 6. (A) Expression levels of m-TOR, p-AKT, p-S6K1, E-cadherin, N-cadherin, Vimentin, and Cyclin D1, normalized against GAPDH control, in OE19. **(B)** Expression levels of m-TOR, E-cadherin, N-cadherin, and Vimentin, normalized against GAPDH control, in OE33.
